# A systematic performance evaluation of clustering methods for single-cell RNA-seq data

**DOI:** 10.12688/f1000research.15666.3

**Published:** 2020-11-16

**Authors:** Angelo Duò, Mark D. Robinson, Charlotte Soneson

**Affiliations:** 1 Institute of Molecular Life Sciences, University of Zurich, Zurich, 8057, Switzerland; 2 SIB Swiss Institute of Bioinformatics, Zurich, 8057, Switzerland

**Keywords:** Clustering, Single-cell RNA-seq, RNA-seq, Benchmarking, Clustering methods

## Abstract

Subpopulation identification, usually via some form of unsupervised clustering, is a fundamental step in the analysis of many single-cell RNA-seq data sets. This has motivated the development and application of a broad range of clustering methods, based on various underlying algorithms. Here, we provide a systematic and extensible performance evaluation of 14 clustering algorithms implemented in R, including both methods developed explicitly for scRNA-seq data and more general-purpose methods. The methods were evaluated using nine publicly available scRNA-seq data sets as well as three simulations with varying degree of cluster separability. The same feature selection approaches were used for all methods, allowing us to focus on the investigation of the performance of the clustering algorithms themselves.

We evaluated the ability of recovering known subpopulations, the stability and the run time and scalability of the methods. Additionally, we investigated whether the performance could be improved by generating consensus partitions from multiple individual clustering methods. We found substantial differences in the performance, run time and stability between the methods, with SC3 and Seurat showing the most favorable results. Additionally, we found that consensus clustering typically did not improve the performance compared to the best of the combined methods, but that several of the top-performing methods already perform some type of consensus clustering.

All the code used for the evaluation is available on GitHub ( https://github.com/markrobinsonuzh/scRNAseq_clustering_comparison). In addition, an R package providing access to data and clustering results, thereby facilitating inclusion of new methods and data sets, is available from Bioconductor ( https://bioconductor.org/packages/DuoClustering2018).

## Introduction

Recent advances in single-cell RNA-seq (scRNA-seq) technologies have enabled the simultaneous measurement of expression levels of thousands of genes across hundreds to thousands of individual cells ^1– 8^. This opens up new possibilities for deconvolution of expression patterns seen in bulk samples, detection of previously unknown cell populations and deeper characterization of known ones. However, computational analyses are complicated by the high variability, low capture efficiency and high dropout rates of scRNA-seq assays ^9– 11^, as well as by strong batch effects that are often confounded by the experimental factor of interest ^12^.

Given a collection of single cells, a common analysis task involves identification and characterization of subpopulations, e.g., cell types or cell states. With lower-dimensional single-cell assays such as flow cytometry, cell type detection is often done manually, by visual inspection of a series of two-dimensional scatter plots of marker pairs (“gating”) and subsequent identification of clusters of cells with specific abundance patterns. With large numbers of markers, such strategies quickly become unfeasible, and they are also likely to miss previously uncharacterized cell populations. Instead, subpopulation detection in higher-dimensional single-cell experiments such as mass cytometry (CyTOF) and scRNA-seq is often done automatically, via some form of clustering. As a consequence, a large number of clustering approaches specifically designed for or adapted to these types of assays are available in the literature ^13^.

While extensive evaluations of clustering methods have been performed for flow and mass cytometry data ^14, 15^, there are to date fewer such studies available for scRNA-seq. The latter is complicated by the large number of different data generation protocols available for scRNA-seq, which in turn has a big effect on the data characteristics. Menon ^16^ specifically evaluated three methods ( Seurat ^17^, WGCNA ^18^ and BackSPIN ^19^), illustrating their different behavior in low and high read depth data. A recent paper ^20^ compared 12 clustering tools on scRNA-seq data sets from the 10x Genomics platform, showing that different methods generally produced clusterings with little overlap. An overview of several different types of clustering algorithms for scRNA-seq data is given by Andrews and Hemberg ^21^.

Here, we extend these initial studies to a broader range of data sets with different characteristics and additionally consider simulated data with different degrees of cluster separability. We evaluate 14 clustering algorithms, including both methods specifically developed for scRNA-seq data, methods developed for other types of single-cell data, and more general approaches, on a total of 12 different data sets. In order to focus on the performance of the clustering algorithms themselves, we use the same preprocessing approach (specifically cell and gene filtering) for all methods, and investigate the impact of the preprocessing separately. In addition to investigating how well the clustering methods are able to recover the true partition if the number of subpopulations is known, we evaluate whether they are able to correctly determine the number of clusters. Further, we study the stability and run time of the methods and investigate whether performance can be improved by generating a consensus partition based on results from multiple individual clustering methods, and the impact of the choice of methods to include in such an aggregation.

We observed large differences in the clustering results as well as in the run times of the different methods. SC3 and Seurat generally performed favorably, with Seurat being several orders of magnitude faster. In addition, Seurat typically achieved the best agreement with the true partition when the number of clusters was the same, while other methods, like FlowSOM, achieved a better agreement with the truth if the number of clusters was higher than the true number. Finally, we show that generally, combining two methods into an ensemble did not improve the performance compared to the best of the individual methods.

Given the high level of activity in methods research for preprocessing, clustering and visualization of scRNA-seq data, it is expected that many new algorithms (or new flavors of existing ones) will be proposed. In order to facilitate re-assessment as new innovations emerge and to provide extensibility to new methods and data sets, all (filtered and unfiltered) data sets as well as all clustering results are accessible via an R/Bioconductor package, leveraging the Bioconductor ExperimentHub framework ( https://bioconductor.org/packages/DuoClustering2018). In addition, the complete code used to run all analyses is available on https://github.com/markrobinsonuzh/scRNAseq_clustering_comparison. The current system uses a Makefile to run a set of R scripts for clustering, summarization and visualization of the results.

## Methods

### Real data sets

Three real scRNA-seq data sets were downloaded from *conquer* ^22^ and used for our evaluations: GSE60749-GPL13112 (here denoted **Kumar** ^23^), SRP073808 ( **Koh** ^24^) and GSE52529-GPL16791 ( **Trapnell** ^25^). These data sets were chosen to represent different degrees of “difficulty” in the clustering task. In particular, the **Trapnell** data set was not generated with the aim of detecting subpopulations, but rather to investigate a continuous developmental trajectory. Nevertheless, it was included in our evaluation as an example of a data set where the phenotype designated as the “true” cluster labels (see below) may not represent the strongest signal present in the data. Table 1 and Supplementary Figure 1 give an overview of all data sets used in this study. For each of the data sets from *conquer*, the gene-level length-scaled TPM values (below referred to as “counts” since they are on the same scale as the raw read counts) and the phenotype were extracted from the MultiAssayExperiment ^27^ object provided by *conquer* and used to create a SingleCellExperiment object. We also estimated transcript compatibility counts (TCCs) for each of these data sets using kallisto ^28, 29^ v0.44, and used these as an alternative to the gene-level count matrix as input to the clustering algorithms.

**Table 1.** Overview of the data sets used in the study.

Data set	Sequencing protocol	# cells	# features	Median total counts per cell	Median # features per cell	# subpopulations	Description	Ref.
**Koh**	SMARTer	531	48,981	1,390,268	14,277	9	FACS purified H7 human embryonic stem cells in different differention stages	24
**KohTCC**	SMARTer	531	811,938	1,391,012	66,086	9	FACS purified H7 human embryonic stem cells in different differention stages	24
**Kumar**	SMARTer	246	45,159	1,687,810	26,146	3	Mouse embryonic stem cells, cultured with different inhibition factors	23
**KumarTCC**	SMARTer	263	803,405	717,438	63,566	3	Mouse embryonic stem cells, cultured with different inhibition factors	23
**SimKumar4easy**	-	500	43,606	1,769,155	29,979	4	Simulation using different proportions of differentially expressed genes	26
**SimKumar4hard**	-	499	43,638	1,766,843	30,094	4	Simulation using different proportions of differentially expressed genes	26
**SimKumar8hard**	-	499	43,601	1,769,174	30,068	8	Simulation using different proportions of differentially expressed genes	26
**Trapnell**	SMARTer	222	41,111	1,925,259	13,809	3	Human skeletal muscle myoblast cells, differention induced by low-serum medium	25
**TrapnellTCC**	SMARTer	227	684,953	1,819,294	66,864	3	Human skeletal muscle myoblast cells, differention induced by low-serum medium	25
**Zhengmix4eq**	10xGenomics GemCode	3,994	15,568	1,215	487	4	Mixture of purified peripheral blood mononuclear cells	5
**Zhengmix4uneq**	10xGenomics GemCode	6,498	16,443	1,145	485	4	Mixture of purified peripheral blood mononuclear cells	5
**Zhengmix8eq**	10xGenomics GemCode	3,994	15,716	1,298	523	8	Mixture of purified peripheral blood mononuclear cells	5

The selected cell phenotype was used to define the “true” partition of cells when evaluating the clustering methods. For the **Kumar** data set, we grouped the cells by the genetic perturbation and the medium in which they were grown. For the **Trapnell** data set we used the time point (after the switch of growth medium) at which the cells were captured, and for the **Koh** data set we used the cell type annotated by the data collectors (obtained through FACS sorting). We note that the definition of the ground truth constitutes an intrinsic difficulty in the evaluation of clustering methods, since it is plausible that there are several different, but still biologically interpretable, ways of partitioning cells in a given data set, several of which can represent equally strong signals. Many public droplet-based data sets contain cell type labels, but these are typically inferred by clustering the cells using the scRNA-seq data, and thus any evaluation based on these labels risks being biased in favor of methods similar to the one used to derive the labels in the first place. By using ground truths that are defined independently of the scRNA-seq assay, we thus avoid artificial inflation of the signal that could result if the truth was derived from the scRNA-seq data itself.

In addition to the data sets from *conquer*, we obtained UMI counts from the Zheng data set ^5^, generated by the 10x Genomics GemCode protocol, from https://support.10xgenomics.com/single-cell-gene-expression/datasets. We downloaded counts for eight pre-sorted cell types (B-cells, naive cytotoxic T-cells, CD14 monocytes, regulatory T-cells, CD56 NK cells, memory T-cells, CD4 T-helper cells and naive T-cells) and combined them into three data sets, with a mix of well-separated (e.g., B-cells vs T-cells) and similar cell types (e.g., different types of T-cells) and uniform and non-uniform cluster sizes. For the data set denoted **Zhengmix4eq**, we combined randomly selected B-cells, CD14 monocytes, naive cytotoxic T-cells and regulatory T-cells in equal proportions (1,000 cells per subpopulation). For the **Zhengmix4uneq** data set, we combined the same four cell types, but in unequal proportions (1,000 B-cells, 500 naive cytotoxic T-cells, 2,000 CD14 monocytes and 3,000 regulatory T-cells). For the **Zhengmix8eq** data set, we combined cells from all eight populations, in approximately equal proportions (400–600 cells per population). For these data sets, we used the annotated cell type (obtained by pre-sorting of the cells) as the true cell label.

### Simulated data sets

Using one subpopulation of the **Kumar** data set as input, we simulated scRNA-seq data with known group structure, using the splatter package ^26^ v1.2.0. We generated three data sets, each consisting of 500 cells, with varying degree of cluster separability. For the **SimKumar4easy** data set, we generated 4 subpopulations with relative abundances 0.1, 0.15, 0.5 and 0.25, and probabilities of differential expression set to 0.05, 0.1, 0.2 and 0.4 for the four subpopulations, respectively. The **SimKumar4hard** data set consists of 4 subpopulations with relative abundances 0.2, 0.15, 0.4 and 0.25, and probabilities of differential expression 0.01, 0.05, 0.05 and 0.08. Finally, the **SimKumar8hard** data set consists of 8 subpopulations with relative abundances 0.13, 0.07, 0.1, 0.05, 0.4, 0.1, 0.1 and 0.05, and probabilities of differential expression equal to 0.03, 0.03, 0.03, 0.05, 0.05, 0.07, 0.08 and 0.1, respectively. The GitHub repository ( https://github.com/markrobinsonuzh/scRNAseq_clustering_comparison) contains a link to a countsimQC report ^30^, comparing the main characteristics of the simulated data sets to those of the underlying **Kumar** data set.

### Data processing

The scater package ^31^ v1.6.3 was used to perform quality control of the data sets. Features with zero counts across all cells, as well as all cells with total count or total number of detected features more than 3 median absolute deviations (MADs) below the median across all cells (on the log scale), were excluded. Depending on the availability of manual annotation, we filtered out cells that were classified as doublets or debris. The scater package was also used to normalize the count values, based on normalization factors calculated by the deconvolution method from the scran package ^32^ v1.6.2, and to perform dimension reduction using PCA ^33^ and t-SNE ^34^. Either the raw feature counts or the log-transformed normalized counts were used as input to the clustering algorithms, following the recommendations by the authors (see Figure 4 for a summary of the input values used for each method).

### Gene filtering

We evaluated three methods for reducing the number of genes provided as input to the clustering methods. For each filtering method, we retained 10% of the original number of genes (with a non-zero count in at least one cell) in the respective data sets. First, we retained only the genes with the highest average expression (log-normalized count) value across all cells (denoted Expr below). Second, we used Seurat ^17^ to estimate the variability of the features and retained only the most highly variable ones (HVG). Finally, we used M3Drop ^35^ to model the dropout rate of the genes as a function of the mean expression level using the Michaelis-Menten equation (M3Drop). The gene-wise Michaelis-Menten constants are computed and log-transformed, and the genes are then ranked by their p-value from a Z-test comparing the gene-wise constants to a global constant obtained by combining all the genes. After filtering, we used scran to renormalize each data set, excluding cells with negative size factors. Supplementary Figure 2 shows the overlap between the retained genes with the different filtering methods, for each of the 12 data sets, and Supplementary Table 1 provides the number of cells retained after each type of filtering.

### Clustering methods

Fourteen clustering methods, publicly available as R packages or scripts, were evaluated in this study (see Table 2 for an overview). We included general-purpose clustering methods, such as hierarchical clustering and K-means, as well as methods developed specifically for scRNA-seq data, such as Seurat and SC3, and methods developed for other types of high-throughput single-cell data ( FlowSOM). The collection of methods include representatives for most types of algorithms commonly used to cluster scRNA-seq data. The type of the underlying clustering algorithm for the different methods is shown in Figure 4.

**Table 2.** Clustering methods.

Method	Description	Reference
ascend (v0.5.0)	PCA dimension reduction (dim=30) and iterative hierarchical clustering	36
CIDR (v0.1.5)	PCA dimension reduction based on zero-imputed similarities, followed by hierarchical clustering	37
FlowSOM (v1.12.0)	PCA dimension reduction (dim=30) followed by self-organizing maps (5×5, 8×8 or 15×15 grid, depending on the number of cells in the data set) and hierarchical consensus meta-clustering to merge clusters	38
monocle (v2.8.0)	t-SNE dimension reduction (initial PCA dim=50, t-SNE dim=3) followed by density-based clustering	25, 39
PCAHC	PCA dimension reduction (dim=30) and hierarchical clustering with Ward.D2 linkage	33, 40
PCAKmeans	PCA dimension reduction (dim=30) and K-means clustering with 25 random starts	33, 41
pcaReduce (v1.0)	PCA dimension reduction (dim=30) and k-means clustering through an iterative process. Stepwise merging of clusters by joint probabilities and reducing the number of dimensions by PC with lowest variance. Repeated 100 times followed consensus clustering using the clue package	42
RaceID2 (March 3, 2017 version)	K-medoids clustering based on Pearson correlation dissimilarities	43
RtsneKmeans	t-SNE dimension reduction (initial PCA dim=50, t-SNE dim=3, perplexity=30) and K-means clustering with 25 random starts	34, 41, 44
SAFE (v2.1.0)	Ensemble clustering using SC3, CIDR, Seurat and t-SNE + Kmeans	45
SC3 (v1.8.0)	PCA dimension reduction or Laplacian graph. K-means clustering on different dimensions. Hierarchical clustering on consensus matrix obtained by K-means	46
SC3svm (v1.8.0)	Using SC3 to derive the clusters for half of the cells, then using a support vector machine (SVM) to classify the rest	46, 47
Seurat (v2.3.1)	Dimension reduction by PCA (dim=30) followed by nearest neighbor graph clustering	17
TSCAN (v1.18.0)	PCA dimension reduction followed by model-based clustering	48

All methods except Seurat allow explicit specification of the desired number of clusters (k). Seurat instead requires a resolution parameter, which indirectly controls the number of clusters. For each data set, we ran each method with a range of k values (from 2 to either 10 or 15, depending on the true number of subpopulations in the data set). We ran Seurat with a range of resolution parameter values, yielding approximately the range of k values evaluated for the other methods. A subset of the methods provide an estimate of the true number of clusters; we record this estimate for comparison with the true number of subpopulations. For each choice of k (or resolution), we ran each method five times, allowing us to investigate the intrinsic stability of the obtained partitions. Note that the data is the same for all five instances, and thus only the stochasticity of the clustering method affects our stability evaluation. All parameter values except for the number of clusters were set to reasonable values following the authors’ recommendations or the respective manuals ( Table 2). Gene and cell filtering within the clustering methods were disabled whenever possible, since these steps were performed in a uniform way during the preprocessing and gene selection steps.

### Evaluation criteria

In order to evaluate how well the inferred clusters recovered the true subpopulations, we used the Hubert-Arabie Adjusted Rand Index (ARI) for comparing two partitions ^49^. The metric is adjusted for chance, such that independent clusterings have an expected index of zero and identical partitions have an ARI equal to 1, and was calculated using the implementation in the mclust R package v5.4. We also used the ARI to evaluate the stability of the clusters, by comparing the partitions from each pair of the five independent runs for each method with a given number of clusters.

We used a normalized Shannon entropy ^50^ to evaluate whether the methods preferentially partitioned the cells into clusters of equal size, or whether they preferred to generate some large and some small clusters. Given proportions *p*_1_, . . . , *p _N_* of cells assigned to each of *N* clusters, the normalized Shannon entropy is defined by

$$\frac{H}{H_{\text{max}}}=-\sum_{i=1}^{N}p_{i}\frac{{\text{log}}_{2}p_{i}}{{\text{log}}_{2}N}.\;\left(1\right)$$

Since the true degree of equality of the cluster sizes varies between data sets, we subtracted the normalized entropy calculated from the true partition to obtain the final performance index.

To evaluate the similarities between the partitions obtained by different methods, we first calculated a consensus partition from the five independent runs for each method, using the clue R package ^51^ v0.3-55. Next, for each data set and each imposed number of clusters, we calculated the ARI between the partitions for each pair of methods, and used hierarchical clustering based on the median of these ARI values across all data sets to generate a dendrogram representing the similarity among the clusters obtained by different methods. To investigate how representative this dendrogram is, we also clustered the methods based on each data set separately, and calculated the fraction of such dendrograms in which each subcluster in the overall dendrogram appeared.

Finally, we investigated whether clustering performance was improved by combining two methods into an ensemble. For each data set, and with the true number of clusters imposed, we calculated a consensus partition for each pair of methods using the clue R package, and used the ARI to evaluate the agreement with the true cell labels. We then compared the ensemble performance to the performances of the two individual methods used to construct the ensemble.

## Results

### Large differences in performance across data sets and methods

**Figure 1.**
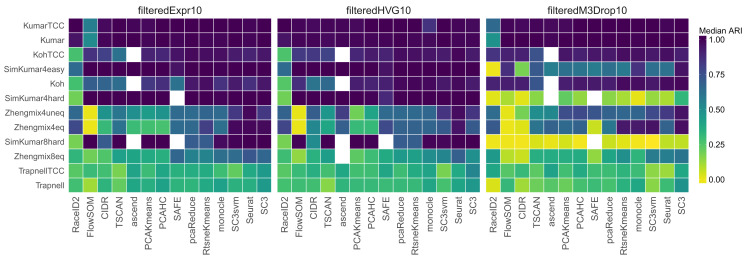
Median ARI scores, representing the agreement between the true partition and the one obtained by each method, when the number of clusters is fixed to the true number. Each row corresponds to a different data set, each panel to a different gene filtering method, and each column to a different clustering method. The methods and the data sets are ordered by their mean ARI across the filterings and data sets. Some methods failed to return a clustering with the correct number of clusters for certain data sets (indicated by white squares).

The 14 methods were tested on real data sets as well as simulations with a varying degree of complexity ( Table 1) and across a range of the number of subpopulations. Focusing on the agreement between the true partitions and the clusterings obtained by imposing the true number of clusters showed a large difference between data sets as well as between methods ( Figure 1; a summary across different numbers of clusters can be found in Supplementary Figure 3).

As expected, excellent performances were achieved for the well-separated data sets with a strong difference between the groups of cells ( **Kumar**, **KumarTCC** and **SimKumar4easy**). When filtering by expression or variability, close to all methods achieved a correct partitioning of the cells in these data sets, while the M3Drop filtering led to a poorer performance for the simulated data set. All methods failed to recover the partition of the cells by time point in the **Trapnell** data sets, where the ARIs were consistently below 0.5. This indicates that there are other, stronger, signals in this data set that dominate the clustering.

We note that the M3Drop filtering consistently led to a worse performance for the simulated data sets, while the performance was more similar to the other filterings for the real data sets, which may indicate that the simulated dropout pattern is not consistent with the one being modeled by the M3Drop package. Due to negative size factor estimates, a larger number of cells had to be excluded in the **Zhengmix** data sets after the M3Drop filtering compared to the expression or HVG filtering (Supplementary Table 1). At most just over 20% of the cells in the expression and HVG filtering and up to approximately 40% of the cells for the M3Drop filtering were excluded, making a direct comparison between the filterings difficult. Furthermore, the genes retained in the M3Drop and expression filterings showed a low degree of overlap in many of the data sets (Supplementary Figure 2). Overall, only small differences were seen between the results for the data sets containing gene abundances and those containing transcript compatibility counts (TCCs).

While none of the methods consistently outperformed the others over the full range of the imposed numbers of clusters in all data sets, SC3 and Seurat often showed the best performance. These methods were also the only ones that achieved a good separation of the cell types in the droplet-based **Zhengmix** data sets, which have a much higher degree of sparsity and a larger number of cells than the other data sets. This is consistent with a previous study ^16^ showing that Seurat performed better than other types of algorithms on data with low read depth. Generally, the performance of Seurat was also not strongly affected by the gene filtering approach (except for the simulated data sets), while other methods, like SAFE, were more sensitive to the choice of input genes for some data sets. FlowSOM showed a poor performance for the true number of clusters (see Supplementary Figure 4 for an illustration, together with a selection of other data set/method combinations with poor ARI values). However, if the number of clusters was increased, the performance of FlowSOM improved considerably, and if the methods instead were compared at the number of clusters that gave the optimal performance for each method, FlowSOM showed a better performance (Supplementary Figure 5). RtsneKmeans, a general-purpose method, showed a higher average performance across the data sets and filterings than many of the clustering algorithms specifically developed for scRNA-seq data. Compared to SC3 and Seurat, RtsneKmeans showed poorer performance for the **SimKumar8hard** and **Zhengmix4uneq** data sets. The subpopulations in these data sets are nested in the t-SNE space, explaining the difficulty in clustering for the K-means algorithm (Supplementary Figure 1).

We also investigated whether the number of detected features per cell differed between the clusters, using a Kruskal-Wallis test ^52^. No strong association was found for the simulated data sets (Supplementary Figure 6), indicating that there is low inherent bias in the clustering algorithms. For most of the real data sets, we found highly significant differences in the number of detected features between cells in different clusters. However, it is unclear whether this represents a technical effect or a biological difference between the cell populations.

### Run times vary widely between methods

**Figure 2.**
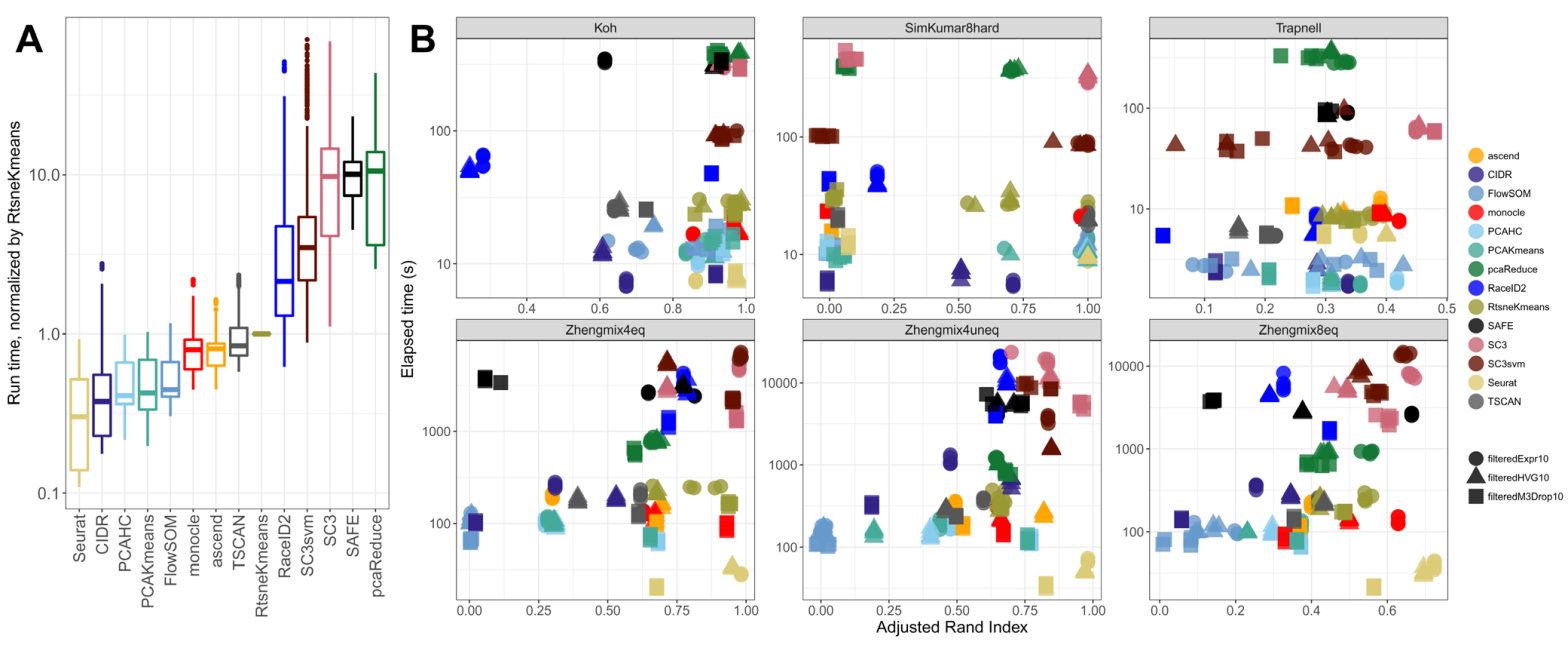
( **A**) Normalized run times, using RtsneKmeans as the reference method, across all data set instances and number of clusters. ( **B**) Run time versus performance (ARI) for a subset of data sets and filterings, for the true number of clusters.

We measured the elapsed time for each run, using a single core and excluding the time to estimate the number of clusters if this was done via a separate function. Since the run times are strongly dependent on the number of features and cells in a data set, we represent them as normalized run times, by dividing with the time required for RtsneKmeans for the same data set ( Figure 2A). Seurat was the fastest method, while pcaReduce, SAFE and SC3 were the slowest, sometimes by a large margin. Clustering only half of the cells with SC3 and predicting the class of the others with a Support Vector Machine ( SC3svm) gave slightly shorter run times than applying the SC3 clustering to all cells. The method could potentially be accelerated by using a lower proportion of cells as a training subset. A detailed overview of the run time and the dependence on the number of clusters is given in Supplementary Figure 7 and Supplementary Figure 8. Apart from SC3 and SC3svm, the imposed number of clusters did not affect the run time.

Plotting the run time versus the ARI for a subset of the data sets (excluding the ones with the strongest signal, where all methods found the correct clusters, and the TCC data sets) ( Figure 2B) further illustrated the variability between the methods. Interestingly, Seurat was generally the fastest method, especially for the droplet-based data sets, but at the same time provided among the best partitionings of the data.

The scalability of the methods was investigated by subsampling the largest data set ( **Zhengmix4uneq**) and plotting the run time as a function of the number of cells (Supplementary Figure 9). The majority of the methods showed a linear increase in run time as a function of the number of cells, while CIDR and RaceID2 scaled worse. The run time of SC3 and SC3svm, and to some extent SAFE, showed more complex patterns since these methods reduce the number of random starts of the Kmeans algorithm drastically if the number of cells exceeds 2,000.

### High stability between clustering runs

**Figure 3.**
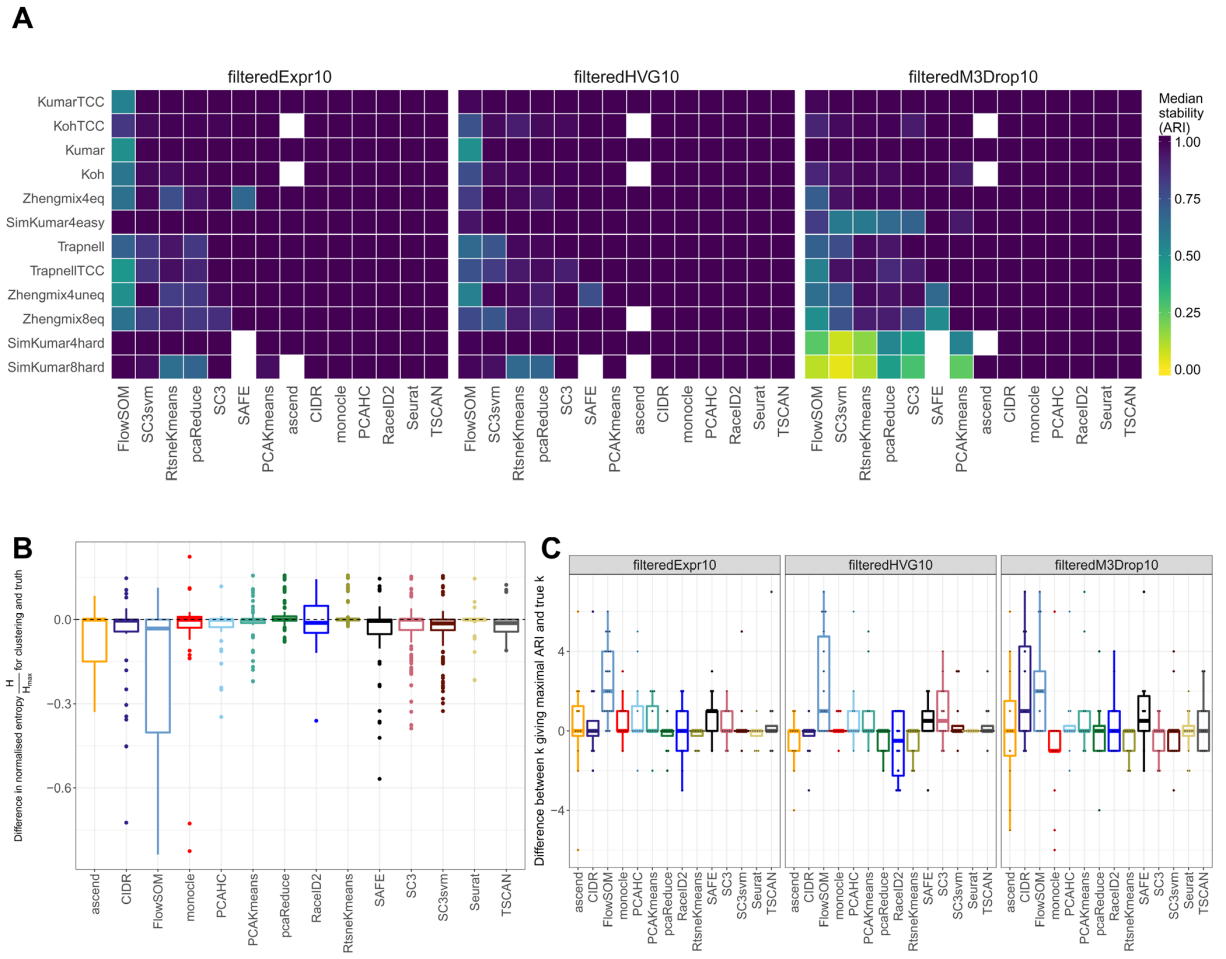
( **A**) Median stability (ARI across different runs on the same data set) for the methods, with the annotated number of clusters imposed. Some methods failed to return a clustering with the correct number of clusters for certain data sets (indicated by white squares). ( **B**) The difference between the normalized entropy of the obtained clusterings and that of the true partitions, across all data sets and for the annotated number of clusters. ( **C**) The difference between the number of clusters giving the maximal ARI and the annotated number of clusters, across all data sets.

Figure 1 illustrated the average performance of each method across the five runs on each data set, for the true number of clusters. By comparing the partitions obtained in the individual runs, we could also obtain a measure of the stability of each method ( Figure 3A).

**Figure 4.**
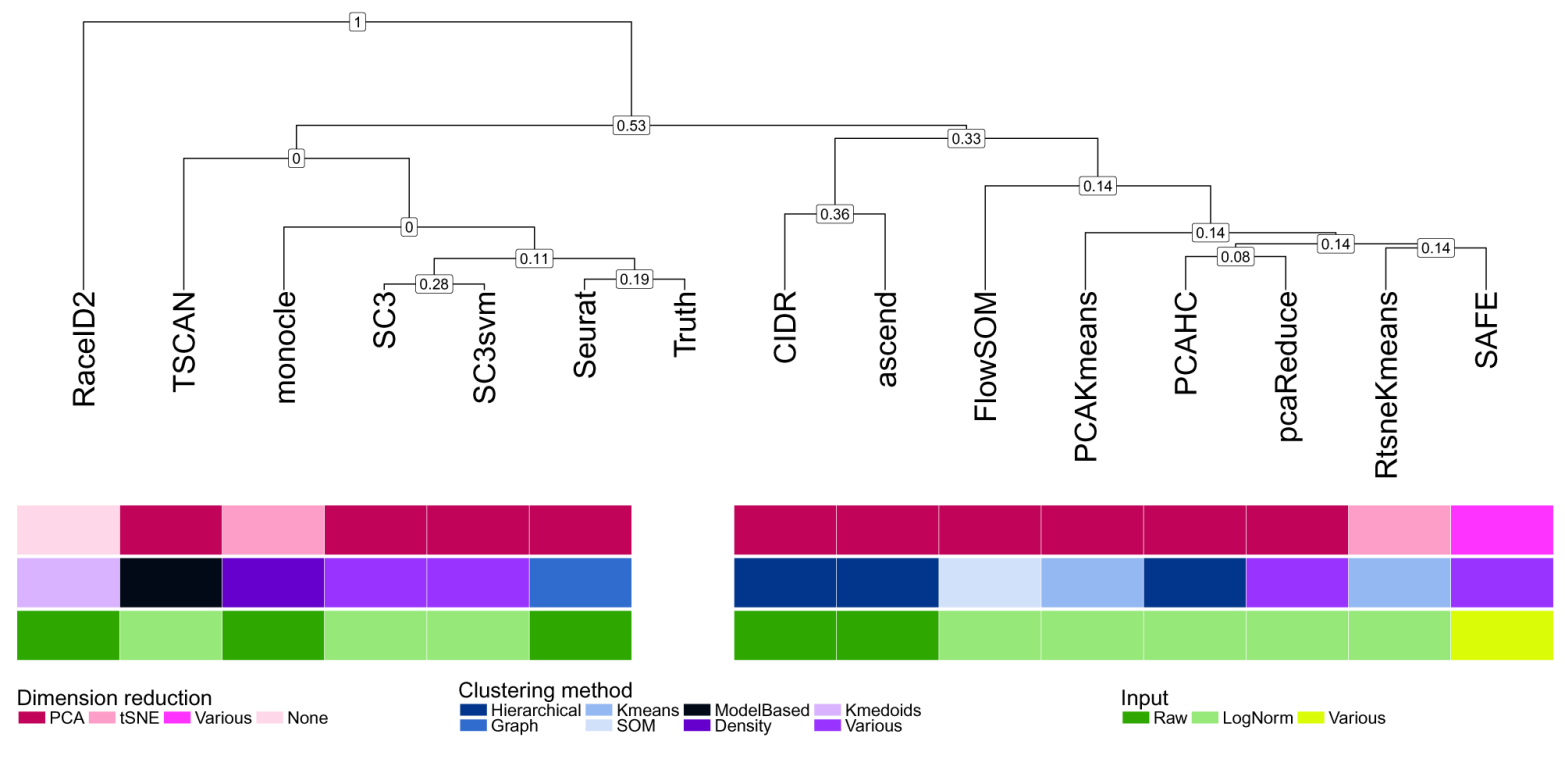
Clustering of the methods based on the average similarity of their partitions across data sets, for the true number of clusters. Numbers on internal nodes indicate the fraction of dendrograms from individual data sets where a particular subcluster was found.

CIDR, monocle, RaceID2, PCAHC, TSCAN, ascend and Seurat returned the same clusters in all five instances for all data sets, while the stability of the other methods depended on the data set. TSCAN and monocle set the random seed to a fixed value internally, which explains the high stability of these methods. Seurat, SC3 and RaceID2 allow the user to set the random seed via an input argument, and we explicitly set this to different values in the five independent runs. Again, the stability was lower for the simulated data sets after gene filtering by M3Drop (note that the same genes were used in all five runs), indicating that the selection of genes may be suboptimal.

A summary of the variability both within and between the different filterings is shown in Supplementary Figure 10. It is worth noting that comparing the performances between the different filtering approaches is difficult for two reasons: first, the variability of the clustering runs for a given filtering might exceed the variation between the filterings, and second, filtering with M3Drop led to the exclusion of a large number of cells in the **Zhengmix** data sets, and these cells can not be used for the comparison. For the stable methods CIDR, TSCAN, ascend and PCAHC, the type of filtering had a relatively large impact on the clustering solutions, and often filtering on the mean gene expression and the gene variability gave more similar clusters than filtering with M3Drop. The stochastic methods showed both a high variability between the individual runs for a given filtering and between runs with different filterings.

### Qualitative differences between cluster characteristics

By computing the Shannon entropy for the various partitions, we obtained a measure of the equality of the sizes of the clusters ( Figure 3B). Since the true degree of cluster size uniformity as well as the number of clusters are different between data sets, we compared the normalized Shannon entropy of the clusterings to that of the true partitions. Thus, a positive value of this statistic indicates that a method tends to produce more equally sized clusters than the true ones, and a negative value instead indicates that the method tends to return more unequal cluster sizes, e.g., one large cluster and a few small ones. Most methods gave cluster sizes that were compatible with the true sizes for most data sets (a statistic close to 0), while especially FlowSOM was more variable, and often tended to group the cells into one large cluster and a few very small ones (see Supplementary Figure 4 for an example). One consequence of this was that FlowSOM often showed higher ARI values for a larger number of clusters, while the performance of many of the other methods decreased with increasing k (Supplementary Figure 3). These methods tended to have more equally sized clusters for larger numbers of clusters than the true number, leading to a higher disagreement between the true classification and the clusterings (the entropy across the range of k is shown in Supplementary Figure 11).

### The optimal number of clusters can differ from the ”true” one

Above, we investigated the performance and stability of the methods when the true number of clusters (the number of different labels in the partitioning considered as the ground truth) was imposed. Whether this number of clusters actually provided the highest ARI value (i.e., the best agreement with the ground truth) mainly depended on the difficulty of the clustering task ( Figure 3C), and the choice of method. No method achieved the best performance at the annotated number of clusters in all the data sets, although generally, the methods reached their maximum performance at or near the annotated number of clusters. The notable exception was FlowSOM, which required a relatively large number of clusters to reach its maximal performance.

SC3, CIDR, ascend, SAFE and TSCAN all have built-in functionality for estimating the optimal number of clusters. In most cases, the estimated number was close to the true one; however, ascend and CIDR had a tendency to underestimate the number of clusters, while SC3 and TSCAN instead tended to overestimate the number (Supplementary Figure 12). The tendency of SC3 to overestimate the cluster number is consistent with a previous publication ^16^. The agreement with the true partition at the estimated number of clusters is shown in Supplementary Figure 13. SC3 is still the best-performing method in this situation.

### Inconsistent degree of similarity between methods

The similarity between each pair of methods was quantified by means of the ARIs for each pair of consensus clusterings (across the five runs of each method for each data set and number of clusters). Figure 4 shows a dendrogram of the methods obtained by hierarchical clustering based on the average ARI values across all data sets for the true number of clusters. The numbers shown at the internal nodes indicate the stability of the subclusters, that is, the fraction of the corresponding dendrograms from the individual data sets where a particular subcluster could be found. In general, the groupings of the methods shown in the dendrogram were unstable across data sets, indicated by the low stability fractions of all subclusters. This is consistent with previous studies showing generally poor concordance that varied across data sets ^20, 45^. Even SC3 and SC3svm had surprisingly different clusterings; in less than a third of the data sets, these two methods showed the most similar clusterings. In addition, no apparent association between the similarity of the clusterings and the type of input or the dimension reduction or underlying type of clustering algorithm was seen ( Figure 4).

### Ensembles often don’t improve clustering performance

**Figure 5.**
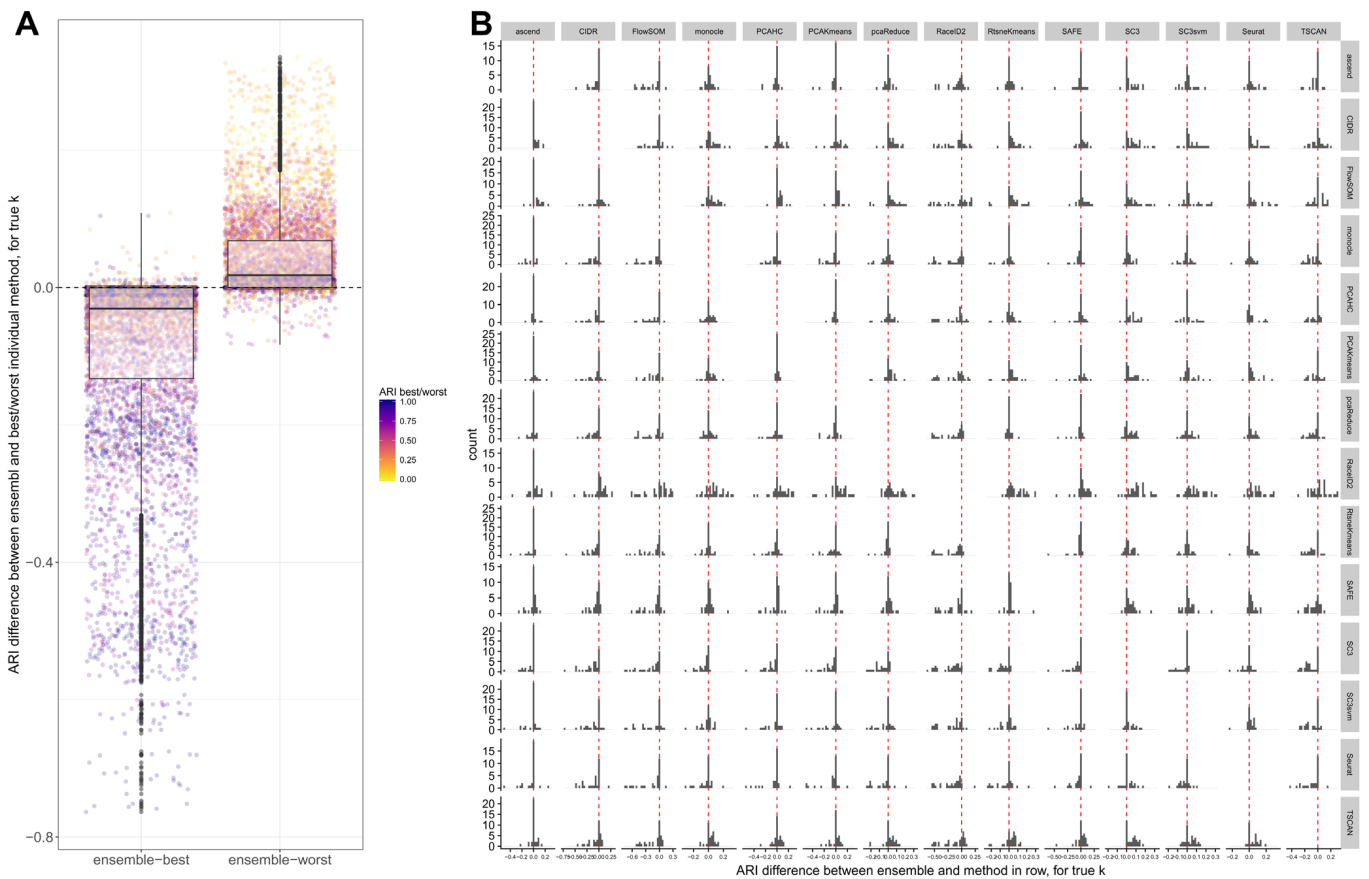
Comparison between individual methods and ensembles. ( **A**) Difference between the ARI of each ensemble and the ARI of the best (left) and worst (right) of the two methods in the ensemble, across all data sets and for the true number of clusters. ( **B**) Difference between the ARI of each ensemble and each of the components, across all data sets and for the true number of clusters. The histogram in row i, column j represents the differences between the ARIs of the ensemble of the methods in row i and column j and the ARI of the method in row i on its own.

Next, we investigated whether we could improve the clustering performance by combining methods into an ensemble. For each pair of methods, we generated a consensus clustering and evaluated its agreement with the true partition using the ARI. In general, the performance of the ensemble was worse than the better of the two combined methods, and better than the worse of the two methods ( Figure 5A), suggesting that we would obtain a better performance by choosing a single good clustering method rather than combining multiple different ones. This is largely consistent with a recent study evaluating the combination of four methods ( SC3, CIDR, Seurat, tSNE+Kmeans), where the ensemble performance was generally on par with the best individual method ^45^. It is still possible that an ensemble method could provide a general improvement over a *given* single method, since it is unlikely that the same method will be the best performing in all conceivable data sets. In fact, among the methods we evaluated, both SC3 and SAFE combine multiple individual methods to achieve the final clustering result. Studying individual combinations in more detail, we observed that combining SC3 or Seurat with almost any other method led to a worse performance than obtained by these methods alone (consistent with the observation that they were among the methods giving the best performance). On the other hand, methods like CIDR, FlowSOM and TSCAN could often be improved by combining them with another method ( Figure 5B).

## Discussion and conclusions

In this study, we have evaluated 14 clustering methods on both real and simulated scRNA-seq data. There were large differences in the ability of the methods to recover the annotated clusters, and performance was also strongly dependent on the degree of separation between the true classes. SC3 and Seurat, two clustering methods developed specifically for single-cell RNA-seq data, delivered the overall best performance, and were the only ones to properly recover the cell types in the droplet-based data sets. There was, however, a large difference in the run time, with SC3 being several orders of magnitude slower than Seurat. Another difference between these two methods is that SC3 includes a method for estimating the number of clusters (which has a tendency towards overestimation), while Seurat will determine the number of clusters based on a resolution parameter set by the user.

The same preprocessing steps and fixed gene sets were used for all clustering methods. This enabled us to investigate the impact of the clustering algorithm itself, rather than entire pipelines or workflows. The selection of the filtering approach had an impact on the majority of the methods and resulted in different clustering solutions. Specifically for the more difficult data sets there was a higher dissimilarity. However, this did not necessarily affect the performances of the methods.

The stability of clustering algorithms can be evaluated by generating perturbed subsamples of the data set and redoing the clusterings. These subsamples can be created in several ways, e.g., by random subsampling with or without replacement, by adding noise to the original data ^53^ or by simulating technical replicates ^54^. Freytag ^20^ showed that SC3, Seurat, CIDR and TSCAN were stable under cell-wise perturbations. In our study, we evaluated the methods with respect to their sensitivity to random starts. Overall, the methods showed a high degree of stability across all data sets, except for the simulated data sets in combination with the M3Drop filtering, where the stochastic methods showed a decrease in stability. This may be due to a disagreement between the mean-dropout relationship in the simulated data and the one assumed by M3Drop, leading to a suboptimal gene selection.

The evaluated methods are based on a broad spectrum of approaches for dimensionality reduction and clustering. We note that the majority of the methods use PCA or PCoA for dimension reduction or Euclidean distances as the distance metric ( ascend allows for other alternatives). Thus, no clear advice on the type of algorithm that is best suited for clustering single-cell RNA-seq data can be made based on our results. In fact, the two best-performing methods, SC3 and Seurat, rely on very different underlying clustering algorithms.

We investigated the impact of changing the imposed number of clusters for the different methods, which revealed that a subset of the methods, in particular FlowSOM, consistently showed a better agreement with the true subpopulations if the number of clusters was increased beyond the true number. The reason for this appears to be that FlowSOM tends to split off a few very small clusters. In addition to the number of clusters, most methods rely on other hyperparameters. In this study, we have fixed these to reasonable values. However, additional investigations into the effect of these hyperparameters on the results would be an interesting direction for future research.

## Data availability

### Underlying data

Bioconductor: DuoClustering2018. https://bioconductor.org/packages/DuoClustering2018.

The R/Bioconductor data package DuoClustering2018 provides full access to the filtered (and unfiltered) data sets, the clustering results from our study and functions for summarizing the performance of different scRNA-seq clustering methods. Additionally, helper functions and descriptions for the evaluation of new methods and data sets are provided.

The package is available under the terms of the GPL (>=2) license.

### Extended data

Zenodo: Supplementary Figures and Tables for Duò *et al.* 2018. https://doi.org/10.5281/zenodo.4165026

This project contains the following extended data:

- 310564e6-a2fe-44c5-8518-698d2633e7df_supp_file_1.pdf (PDF file containing Supplementary Figures 1–13 and Supplementary Table 1)

Data are available under the terms of the CC-BY 4.0 license.

Zenodo: Archived R scripts as at time of publication. https://doi.org/10.5281/zenodo.1314743

This project contains the R scripts used to run the analyses presented in this article. Material from this repository are available under the terms of the MIT license.

GitHub: Latest version of R scripts used for analysis. https://github.com/markrobinsonuzh/scRNAseq_clustering_comparison.

This repository contains the latest version of the R scripts used to run the analyses presented in this article. Material from this repository are available under the terms of the MIT license.
