# Supplementary material for: A systematic performance evaluation of clustering methods for single-cell RNA-seq data
Source: F1000Res. 2020 Nov 16;7:1141. Originally published 2018 Jul 26. [Version 3] doi: 10.12688/f1000research.15666.3 (PMC6134335; doi:10.12688/f1000research.15666.3)
Supplement: Peer review history

# Peer review: Reviewer response for version 2

**DOI:** 10.5256/f1000research.17687.r38139

**Published:** 2018-09-11

**Authors:** Saskia Freytag

**Affiliations:** 1 Department of Medical Biology , University of Melbourne, Parkville, Vic, Australia

I am satisfied with the changes the authors have made.

 Furthermore, I want to commend the authors for making the data accessible through an R package to facilitate further benchmarking of single cell clustering methods.

Is the work clearly and accurately presented and does it cite the current literature?

Yes

If applicable, is the statistical analysis and its interpretation appropriate?

Yes

Are all the source data underlying the results available to ensure full reproducibility?

Yes

Is the study design appropriate and is the work technically sound?

Partly

Are the conclusions drawn adequately supported by the results?

Yes

Are sufficient details of methods and analysis provided to allow replication by others?

Yes

Reviewer Expertise:

NA

I confirm that I have read this submission and believe that I have an appropriate level of expertise to confirm that it is of an acceptable scientific standard.

# Peer review: Reviewer response for version 2

**DOI:** 10.5256/f1000research.17687.r38138

**Published:** 2018-09-10

**Authors:** Jean Fan

**Affiliations:** 1 Department of Biomedical Informatics, Harvard Medical School, Boston, MA, USA; 2 Department of Chemistry and Chemical Biology, Harvard University, Cambridge, MA, USA

The authors have thoroughly addressed my comments. Based on the new revised results, I have the following minor comment:

 - The authors note that "We found substantial differences in the performance, run time and stability between the methods, with SC3 and Seurat showing the most favorable results."

 Please clarify what metric(s) you are using to assess "most favorable results" ie. "most favorable results in terms of overall accuracy in cell-type identification", "in terms of run time", "in terms of stability", etc. Based on your new Supplemental Figure 9, it seems that SC3 would not be favorable in terms of runtime/scalability. 

 With this minor comment addressed, I find this article now suitable for indexing in F1000 and look forward to the authors' future follow-up work.

Is the work clearly and accurately presented and does it cite the current literature?

Partly

If applicable, is the statistical analysis and its interpretation appropriate?

Partly

Are all the source data underlying the results available to ensure full reproducibility?

Yes

Is the study design appropriate and is the work technically sound?

Partly

Are the conclusions drawn adequately supported by the results?

Partly

Are sufficient details of methods and analysis provided to allow replication by others?

Yes

Reviewer Expertise:

NA

I confirm that I have read this submission and believe that I have an appropriate level of expertise to confirm that it is of an acceptable scientific standard.

# Response

**Published:** 2018-09-21

**Authors:** Angelo Duò

**Affiliations:** University of Zurich, Switzerland

Thank you for your comment. SC3 and Seurat show the highest accuracy when the true number of clusters is used as the input parameter. Also, SC3 has the highest median ARI when the estimated number of clusters is imposed. Similarly, using the number of clusters that gives the maximum performance, SC3 evaluates best. These two methods have both the lowest variability within and between the different filterings. Seurat is stable with regards to random starts and at least for the datasets filtered on the average expression and the variability. Finally, whereas Seurat was generally the fastest method, we agree that SC3 is currently not favorable in terms of the runtime or scalability.

# Peer review: Reviewer response for version 1

**DOI:** 10.5256/f1000research.17093.r36545

**Published:** 2018-08-03

**Authors:** Saskia Freytag

**Affiliations:** 1 Department of Medical Biology , University of Melbourne, Parkville, Vic, Australia

Overview

 The authors present comprehensive benchmarking of clustering tools in R on real and simulated single-cell RNA-seq datasets. Their work includes performance, stability and run time analysis. Furthermore, they also investigate whether combining results from different methods increases performance.

 Major comments

  

- Throughout the entire manuscript the authors should make it clear that only clustering tools available in R were investigated. This is important, as there are quite a number of popular python applications for clustering of single cell RNA-seq data available.
- Like Jean Fan, I am concerned about the appropriateness of the Trapnell et al. dataset and the Zheng et al. 10x datasets. Furthermore for the Zheng et al. dataset, I would like to know why the authors did not use all 10 pre-sorted cell populations available? Furthermore, how did the authors choose which cell populations to combine for their Zhengmix4 and Zhengmix8 datasets?

Minor comments

- The authors show nicely that Seurat is not very strongly affected by gene filtering. Could this be a result of its clustering approach being based on the 500 most variable genes?
- On page 7 in the paragraph “Run Times vary widely between methods” the authors use Adjusted Rand Index instead of its already introduced abbreviation 
- Could the size of Figure 5 be increased?
- Why did some methods get raw and some methods log-transformed normalized counts?
- Consider changing Supplementary Figure 2 to a visual representation that represents size differences between sets, like UpSetR plots.
- On page 10 the authors say: ”In addition, no apparent association between the similarity of the clusterings and the type of input or dimension reduction or underlying type of clustering algorithm was found.” Could the authors explain in more detail how this analysis was performed.
- On page 6, the authors speculate that there are stronger signals that dominate clustering in the Trapnell et al dataset that are not time points. What could these be? Have the authors investigated cell cycle?

Is the work clearly and accurately presented and does it cite the current literature?

Yes

If applicable, is the statistical analysis and its interpretation appropriate?

Yes

Are all the source data underlying the results available to ensure full reproducibility?

Yes

Is the study design appropriate and is the work technically sound?

Partly

Are the conclusions drawn adequately supported by the results?

Yes

Are sufficient details of methods and analysis provided to allow replication by others?

Yes

Reviewer Expertise:

NA

I confirm that I have read this submission and believe that I have an appropriate level of expertise to confirm that it is of an acceptable scientific standard, however I have significant reservations, as outlined above.

# Response

**Published:** 2018-08-31

**Authors:** Charlotte Soneson

**Affiliations:** Friedrich Miescher Institute for Biomedical Research, Switzerland

*Thank you for reviewing our manuscript and for your constructive comments. Below are point-by-point responses to the individual comments.*

 Throughout the entire manuscript the authors should make it clear that only clustering tools available in R were investigated. This is important, as there are quite a number of popular python applications for clustering of single cell RNA-seq data available.

*This has been clarified in the Abstract as well as in the Methods part of the text. Some of the most widely used clustering methods implemented in Python (e.g., scanpy) implement the same or similar clustering methods as those evaluated in this study, and could thus be considered to be implicitly investigated. Also, the evaluation system we provide (via the code in the GitHub repository and the associated data package) is not strictly limited to methods implemented in R; other methods can be included e.g. using system() calls.*

 Like Jean Fan, I am concerned about the appropriateness of the Trapnell et al. dataset and the Zheng et al. 10x datasets. Furthermore for the Zheng et al. dataset, I would like to know why the authors did not use all 10 pre-sorted cell populations available? Furthermore, how did the authors choose which cell populations to combine for their Zhengmix4 and Zhengmix8 datasets?

*We agree that the Trapnell data set was not generated with the purpose of finding cell types - however, we still find it useful to illustrate the performance of the methods in a data set where the “true clusters” (defined as the time point at which the cells where collected) do not represent the main/strongest signal in the data (see e.g. the t-SNE plots in Supplementary Figure 1). We have clarified this in the “Methods-Real data sets” section of the revised paper.*

**

*For the Zhengmix data sets, our aim was to generate data sets with a mix of well-separated (e.g., B-cells vs T-cells)  and similar cell types (e.g., different types of T-cells). In addition, we wanted to investigate if the number of cell populations and/or the equality of the population sizes had an impact on the performance. The included cell type combinations were selected to allow us to address these questions; however, given the richness of this data set, there are certainly many more possible combinations to explore. We have expanded the description in the “Methods-Real data sets” section a bit to highlight these goals.*

 The authors show nicely that Seurat is not very strongly affected by gene filtering. Could this be a result of its clustering approach being based on the 500 most variable genes?

*In all our investigations, we preselect the genes that are used as input for each clustering algorithm using three different variable selection methods, and internal variable selection or filtering steps are disabled. Specifically, for Seurat we perform the PCA using all the genes remaining after our filtering, and the clustering is then performed in the principal component space. Thus, the stability of Seurat should be affected in the same way as that of the other methods by the selection of variables.*

 On page 7 in the paragraph “Run Times vary widely between methods” the authors use Adjusted Rand Index instead of its already introduced abbreviation

*Thanks for noticing this, we now use the abbreviation also here.*

 Could the size of Figure 5 be increased?

*We have increased the size of Figure 5B.*

 Why did some methods get raw and some methods log-transformed normalized counts?

*The methods are based on different distributional assumptions and underlying models, affecting the type of values that are most suitably used as input. We followed the recommendations of the authors of the respective methods, and the type of input used for each method is summarized in Figure 4.*

 Consider changing Supplementary Figure 2 to a visual representation that represents size differences between sets, like UpSetR plots.

*We have replaced the Venn diagrams in Supplementary Figure 2 with UpSet plots.*

 On page 10 the authors say: ”In addition, no apparent association between the similarity of the clusterings and the type of input or dimension reduction or underlying type of clustering algorithm was found.” Could the authors explain in more detail how this analysis was performed.

*This conclusion is drawn based on Figure 4, where no association between the clustering of methods by cluster similarity and any of the method characteristics can be seen. This has been clarified in the “Results-Inconsistent degree of similarity between methods” section of the revised paper.*

 On page 6, the authors speculate that there are stronger signals that dominate clustering in the Trapnell et al dataset that are not time points. What could these be? Have the authors investigated cell cycle?

*We have not explicitly investigated the interpretation of the strongest signal in the Trapnell data set. However, Supplementary Figure 1 suggests that the annotation that we used to define the “true” clusters (the time at which the cells were collected) does not fully explain the grouping of the cells in the t-SNE visualization (in particular, the T12 and T24 groups are intermingled). As noted above, the main purpose of including this data set was to investigate the behaviour of the various methods in a data set where the clusters were less apparent.*

# Peer review: Reviewer response for version 1

**DOI:** 10.5256/f1000research.17093.r36544

**Published:** 2018-07-27

**Authors:** Jean Fan

**Affiliations:** 1 Department of Biomedical Informatics, Harvard Medical School, Boston, MA, USA; 2 Department of Chemistry and Chemical Biology, Harvard University, Cambridge, MA, USA

Overview

 Duo et al compare multiple single-cell RNA-seq clustering approaches on real and simulated single-cell RNA-seq datasets. 

 Major comments

 - Quite a number of single-cell RNA-seq datasets are available for benchmarking but only a few were explored here. While an exhaustive interrogation of all single-cell RNA-seq datasets available is beyond the scope of this paper, it would be worthwhile for the readers if the authors could comment briefly on the appropriateness of the datasets used here in terms of their cell-type diversity or other factors that may impact benchmarking. As the authors note, a method's performance is inherently tied to the degree to which the tested subpopulations are truly  (or artificially) transcriptionally distinct. In particular, I am concerned about the appropriateness of the Trapnell dataset, as it was originally intended for pseutotime/trajectory inference and may not even contain discrete transcriptional subpopulations. The poor performance as noted in Figure 1 for this dataset may simply arise from different methods cutting along this continuous trajectory in different ways. Similarly, for the Zheng 10x datasets, since each cell-type was sorted and sequenced separately, there is inevitably some degree of confounding of cell-type specific effects with batch effects that could make clustering much easier. 

 - As datasets get bigger, the scalability of each method will be an important consideration. The authors provide a preliminary look into this via the different run time of each method in Figure 2, but how this run time depends on the number of cells is unclear. Readers will be interested in whether some methods scale better than others. It is worth having an additional figure of run time as a function of number of cells (via downsampling cells and then extrapolating to larger datasets) to fully capture the scalability of each method. 

 - With regard to the stability between cluster runs, some methods may internally set various random seeds to ensure reproducibility. Please double check that the stability observed in Figure 3 is not simply the result of which methods uses random seeds. If a method does use an (or likely multiple) internal random seed, the seed must be changed to accurately assess stability. 

 Minor comments

 - There are quite a number of single-cell RNA-seq clustering approaches and the list keeps growing (https://github.com/seandavi/awesome-single-cell). Only a fraction is represented in this comparison. While an exhaustive comparison of all methods is beyond the scope of this paper, the authors should comment briefly on how these particular 12 clustering algorithms were chosen.

 - While nearly all methods assessed use dimensionality reduction as a first step, it is unclear why some were allowed to reduce to 30 dimensions while others 50. It seems that particularly as datasets get larger with presumably more cell-types captured in each datasets, we will likely want to increase the number of PCs to fully capture the variation present in the data. While the authors have left the investigation into the effects of the number of PCs to future research, they should briefly note the reason for the choice of PCs used for each method.

Is the work clearly and accurately presented and does it cite the current literature?

Partly

If applicable, is the statistical analysis and its interpretation appropriate?

Partly

Are all the source data underlying the results available to ensure full reproducibility?

Yes

Is the study design appropriate and is the work technically sound?

Partly

Are the conclusions drawn adequately supported by the results?

Partly

Are sufficient details of methods and analysis provided to allow replication by others?

Yes

Reviewer Expertise:

NA

I confirm that I have read this submission and believe that I have an appropriate level of expertise to confirm that it is of an acceptable scientific standard, however I have significant reservations, as outlined above.

# Response

**Published:** 2018-08-31

**Authors:** Charlotte Soneson

**Affiliations:** Friedrich Miescher Institute for Biomedical Research, Switzerland

*Thank you for reviewing our manuscript and for your constructive comments. Below are point-by-point responses to the individual comments.*

 Quite a number of single-cell RNA-seq datasets are available for benchmarking but only a few were explored here. While an exhaustive interrogation of all single-cell RNA-seq datasets available is beyond the scope of this paper, it would be worthwhile for the readers if the authors could comment briefly on the appropriateness of the datasets used here in terms of their cell-type diversity or other factors that may impact benchmarking. As the authors note, a method's performance is inherently tied to the degree to which the tested subpopulations are truly  (or artificially) transcriptionally distinct. In particular, I am concerned about the appropriateness of the Trapnell dataset, as it was originally intended for pseutotime/trajectory inference and may not even contain discrete transcriptional subpopulations. The poor performance as noted in Figure 1 for this dataset may simply arise from different methods cutting along this continuous trajectory in different ways. Similarly, for the Zheng 10x datasets, since each cell-type was sorted and sequenced separately, there is inevitably some degree of confounding of cell-type specific effects with batch effects that could make clustering much easier.

*There is indeed a large (and increasing) number of public scRNA-seq data sets available, generated with many different types of protocols. However, the main issue (especially with droplet-based data sets) is that no independent annotation of the cells is available, which implies that they are not suitable for unbiased benchmarking like we are doing here. Many public droplet-based data sets do contain “cell type labels”, but these are typically inferred by clustering the cells based on the scRNA-seq data itself, and thus any evaluation risks being biased in favor of methods similar to the one used to derive the labels in the first place. This is the main reason behind the selection of these data sets. We agree that the Trapnell data set was not generated with the purpose of finding cell types - however, we still find it useful to illustrate the performance of the methods in a data set where the “true clusters” (defined as the time point at which the cells where collected) do not represent the main/strongest signal in the data (see e.g. the t-SNE plots in Supplementary Figure 1). For the Zheng data set, it’s true that there could be confounding with batch effects, and ambiguous cells may be excluded, which would also make clusters more distinct. For our Zhengmix data sets, we therefore included both very different (e.g., B-cells and T-cells) and more similar (e.g., different types of T-cells) cell types (Supplementary Figure 1). We have expanded the discussion in the “Methods-Real data sets” section of the revised paper to clarify these issues.*

 As datasets get bigger, the scalability of each method will be an important consideration. The authors provide a preliminary look into this via the different run time of each method in Figure 2, but how this run time depends on the number of cells is unclear. Readers will be interested in whether some methods scale better than others. It is worth having an additional figure of run time as a function of number of cells (via downsampling cells and then extrapolating to larger datasets) to fully capture the scalability of each method.

*Thanks for pointing this out. We have included a plot illustrating the scalability, investigated by downsampling of the largest data set, in Supplementary Figure 9.*

 With regard to the stability between cluster runs, some methods may internally set various random seeds to ensure reproducibility. Please double check that the stability observed in Figure 3 is not simply the result of which methods uses random seeds. If a method does use an (or likely multiple) internal random seed, the seed must be changed to accurately assess stability.

*Two of the methods (TSCAN and monocle) set random seeds internally and do not allow these to be changed by the user. Other methods (SC3, Seurat and RaceID2) set a random seed but let the user specify it. For these methods, we explicitly set the random seed to different values in the five runs. We have clarified this in the “Results-High stability between clustering runs” section of the revised text.*

 There are quite a number of single-cell RNA-seq clustering approaches and the list keeps growing (https://github.com/seandavi/awesome-single-cell). Only a fraction is represented in this comparison. While an exhaustive comparison of all methods is beyond the scope of this paper, the authors should comment briefly on how these particular 12 clustering algorithms were chosen.

*The methods were chosen to represent the most common types of algorithms used for clustering of scRNA-seq data. We have tried to include the most widely used methods, but also to include methods from tangential fields as well as more traditional clustering methods to serve as a baseline. We have clarified this in the text.*

 While nearly all methods assessed use dimensionality reduction as a first step, it is unclear why some were allowed to reduce to 30 dimensions while others 50. It seems that particularly as datasets get larger with presumably more cell-types captured in each datasets, we will likely want to increase the number of PCs to fully capture the variation present in the data. While the authors have left the investigation into the effects of the number of PCs to future research, they should briefly note the reason for the choice of PCs used for each method.

*We extracted 50 principal components for the methods that performed an additional dimension reduction (by t-SNE), and 30 principal components for methods where the clustering was done in the principal component space. The only exception was FlowSOM; this was unintentional and has been harmonized in the revised version to use the same number of PCs as the rest of the methods.*
